# In-Depth Assessment of Within-Individual and Inter-Individual Variation in the B Cell Receptor Repertoire

**DOI:** 10.3389/fimmu.2015.00531

**Published:** 2015-10-12

**Authors:** Jacob D. Galson, Johannes Trück, Anna Fowler, Márton Münz, Vincenzo Cerundolo, Andrew J. Pollard, Gerton Lunter, Dominic F. Kelly

**Affiliations:** ^1^Oxford Vaccine Group, Department of Paediatrics, The NIHR Oxford Biomedical Research Center, University of Oxford, Oxford, UK; ^2^Wellcome Trust Centre for Human Genetics, University of Oxford, Oxford, UK; ^3^Paediatric Immunology, University Children’s Hospital, Zürich, Switzerland; ^4^Medical Research Council Human Immunology Unit, Weatherall Institute of Molecular Medicine, Oxford, UK

**Keywords:** B cell, antibody diversity, immunoglobulin repertoire, B cell receptor repertoire, VDJ recombination, genetic variation, immunoglobulin gene, reproducibility

## Abstract

High-throughput sequencing of the B cell receptor (BCR) repertoire can provide rapid characterization of the B cell response in a wide variety of applications in health, after vaccination and in infectious, inflammatory and immune-driven disease, and is starting to yield clinical applications. However, the interpretation of repertoire data is compromised by a lack of studies to assess the intra and inter-individual variation in the BCR repertoire over time in healthy individuals. We applied a standardized isotype-specific BCR repertoire deep sequencing protocol to a single highly sampled participant, and then evaluated the method in 9 further participants to comprehensively describe such variation. We assessed total repertoire metrics of mutation, diversity, VJ gene usage and isotype subclass usage as well as tracking specific BCR sequence clusters. There was good assay reproducibility (both in PCR amplification and biological replicates), but we detected striking fluctuations in the repertoire over time that we hypothesize may be due to subclinical immune activation. Repertoire properties were unique for each individual, which could partly be explained by a decrease in IgG2 with age, and genetic differences at the immunoglobulin locus. There was a small repertoire of public clusters (0.5, 0.3, and 1.4% of total IgA, IgG, and IgM clusters, respectively), which was enriched for expanded clusters containing sequences with suspected specificity toward antigens that should have been historically encountered by all participants through prior immunization or infection. We thus provide baseline BCR repertoire information that can be used to inform future study design, and aid in interpretation of results from these studies. Furthermore, our results indicate that BCR repertoire studies could be used to track changes in the public repertoire in and between populations that might relate to population immunity against infectious diseases, and identify the characteristics of inflammatory and immunological diseases.

## Introduction

An effective humoral immune response is in part dependent on having a diversity of B cells with different B cell receptors (BCRs) capable of recognizing and binding to many different antigens. The sum of all B cells with distinct BCRs is termed the BCR repertoire, and in humans has the theoretical potential to reach a size of up to 10^11^ unique variants (1). BCRs consist of paired heavy and light chains, with primary diversity generated by the somatic recombination of V, D (heavy chain only), and J gene segments during B cell development to form the functional genes (2). Further diversity is introduced by the random addition and deletion of nucleotides at the junctions of the gene segments. Upon B cell activation and proliferation, there is a secondary diversification step mediated by somatic hypermutation, and selection of B cells with increasing affinity for the antigen (3). Although the BCR repertoire is often considered as a single entity, it is actually a mixture of different B cell subpopulations, each of which may have a distinct repertoire structure (4, 5).

The vast diversity of the BCR repertoire has made it difficult to study, but next-generation sequencing (NGS) technology now makes it possible to capture a high-resolution snapshot of the circulating BCR repertoire in humans. Obtaining information on paired heavy and light chains is technically challenging (6), so studies of the BCR repertoire generally focus on the heavy chain, which is the most variable, and most important for determining antigen-binding specificity (7). BCR repertoire analysis has been used to increase understanding of the fundamental properties of B cells, including developmental processes (8), and responses to antigen (4, 9–13). In addition, a number of clinical applications are beginning to emerge, including identification of autoimmune irregularities (14), monitoring of B cell lymphoma minimal disease residue (15, 16), disease diagnostics (17), and the rapid identification of monoclonal antibody sequences (18). These studies generally monitor global features of the BCR repertoire, such as diversity, mutation levels, isotype subclass usage, and VDJ segment usage frequency as well as identifying specific B cell clones. Identifying B cells arising from the same clonal origin was initially conducted based on identifying common VDJ segment usage (15), but there is now a move toward incorporating complementarity-determining region (CDR) three amino acid (AA) sequence identity into the definition (8, 17), which forms at the junction of VDJ joining and is the most important region for determining antigen-binding properties (19).

B cell samples for repertoire studies in humans are usually obtained from peripheral blood as this is an easy to sample compartment, but it is not possible to extract all peripheral blood from humans, and some B cells may be present in different compartments. This means that the entire repertoire cannot be sampled, so instead a representative sample is taken. It is, therefore, important to quantify exactly what proportion of the repertoire is being sampled, and how repeatable the sampling is. This is particularly pertinent for clinical applications where it is necessary to have a highly repeatable measure to detect the clones of interest. As BCR repertoire sequencing is a young technology, such repeatability studies have been done in mice (20), but not exhaustively in humans, who have a much larger repertoire (1). Many studies of the BCR repertoire also assess changes in various features of the repertoire over time in response to certain interventions (9, 11–13). However, there is relatively little known about how the much the repertoire naturally fluctuates over time in the absence of any specific intervention, making it difficult to discern natural fluctuations from intervention-induced fluctuations. Furthermore, as the total number of healthy individuals who have had their repertoire sequenced remains small, there is not a clear consensus of what can be construed as a “normal” repertoire. It is uncertain to what extent similar B cell clones can be found in multiple individuals (the public repertoire) not undergoing a similar immune stimulus, and whether sharing is just due to chance, or due to historic expansion of similar B cells in different individuals from a common antigen and thus have some clinical significance (4, 11, 17, 21).

**Figure 1:**
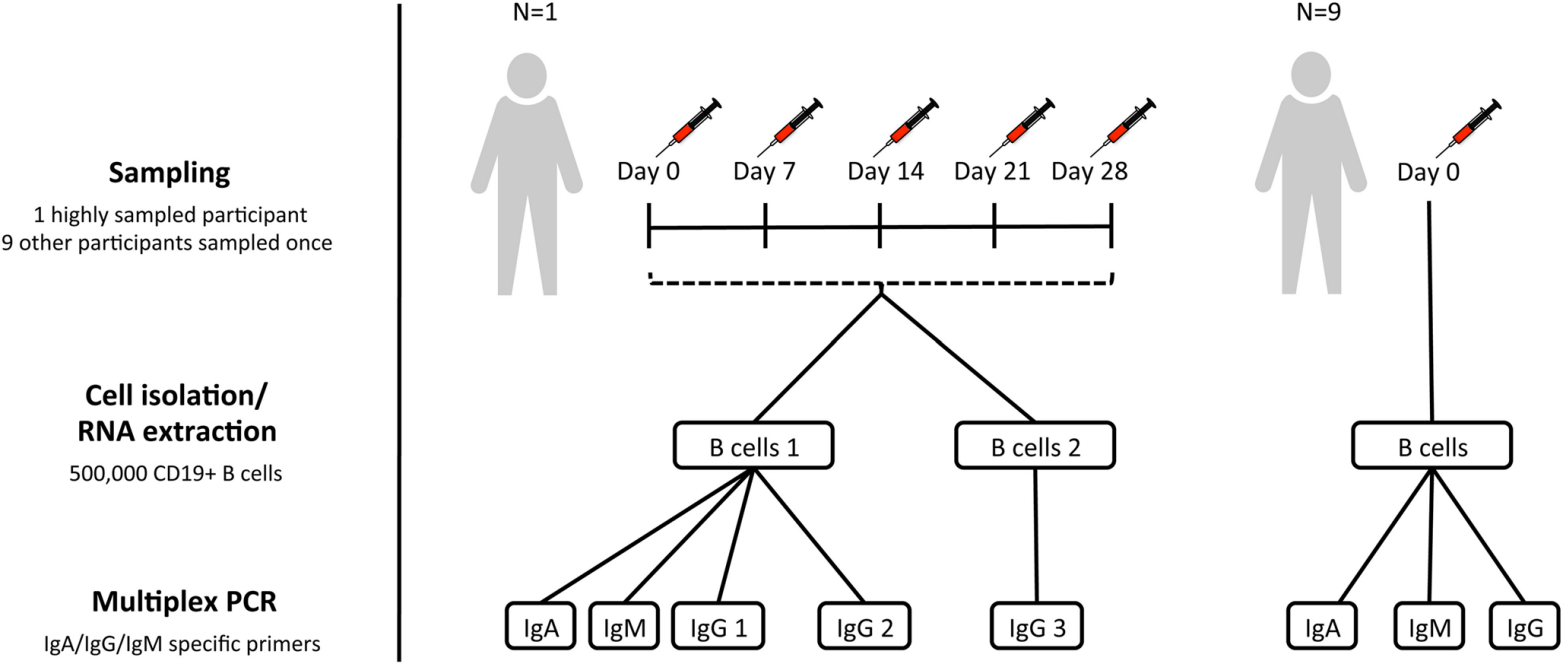
**Sampling scheme**. For assessment of within-individual variation, one participant (participant AF01) had blood sampled at five timepoints 1 week apart (temporal replicates). For this participant, two aliquots of B cells were taken at each timepoint to give biological replicates. IgA, IgG, and IgM isotype-specific PCRs were all conducted for one of the biological replicates, but only IgG for the second biological replicate (IgG 3). Additionally, for this participant, IgG PCR replicates were also conducted at each timepoint for one of the biological replicates (IgG 2). For assessment of inter-individual variation, nine additional participants were sampled at a single timepoint, and had IgA, IgG, and IgM PCRs conducted.

We sought to shed light on these questions by carrying out repeat sampling from a single individual to assess the robustness of a BCR repertoire sequencing protocol, and also to determine how the repertoire changed over time in the absence of any intervention (Figure 1). Furthermore, sequencing of the BCR repertoire from nine additional individuals was conducted to determine how variable the repertoire was between them, and to interrogate properties of the public repertoire.

## Materials and Methods

### Sample Collection

Subjects were recruited with informed consent in accordance with the Declaration of Helsinki, and under approval from the Northampton Research Ethics Committee (13/EM/0036). For the assessment of inter-individual variation, 10 participants had blood sampled at a single timepoint. In addition, to assess within-individual variation, and repeatability of the protocol, there was a single highly sampled participant, who had blood sampled on five consecutive weeks (days 0, 7, 14, 21, and 28) to give temporal replicates (Figure 1). Blood was transferred to a heparinized tube for processing within 4 h of collection.

### B Cell Isolation

PBMC’s were first isolated from heparinized blood by density-gradient centrifugation over lymphoprep (Axis-Shield Diagnostics). Magnetic-activated cell sorting was then used to isolate B cells, using the AutoMACS Pro cell separator (Miltenyi Biotec), and CD19^+^ microbead kit. B cells were counted using a hemocytometer (Neubauer), and split into aliquots each containing 500,000 cells. Sorted B cells were resuspended in RLT buffer (Qiagen) and frozen at −80°C prior to repertoire sequencing. For each participant, a single 500,000 B cell aliquot was used for repertoire sequencing. An additional 500,000 B cell aliquot was also taken for the highly sampled participant to give biological replicates.

### Repertoire Sequencing

RNA was extracted from sorted cells using the RNeasy Mini Kit (Qiagen), and reverse transcription performed using SuperScript III (Invitrogen), and random hexamer primers (42°C for 60 min, 95°C for 10 min). BCR heavy chain genes were amplified using the Multiplex PCR kit (Qiagen) with V family specific forward primers, and in separate reactions with either IgA, IgG, or IgM specific reverse primers (22) (94°C for 15 min, 30 cycles of 94°C for 30 s, 58°C for 90 s and 72°C for 30 s, and 72°C for 10 min). For the biological replicates, only IgG reactions were performed, but for all other samples, IgA, IgG, and IgM reactions were all carried out. In addition, for one of the aliquots at each timepoint from the highly sampled participant, the IgG PCR was repeated to give PCR replicates. PCR amplicons were gel-extracted, purified, and quantified using a Qubit fluorometer (Invitrogen). Samples were then A-tailed and adaptor ligated prior to size selection and amplification for sequencing on the MiSeq using the 2 × 300 bp paired-end chemistry (Illumina). Samples were multiplexed in batches of 50 for sequencing, and indexed using the Illumina tags.

### Raw Sequence Processing

Paired-end reads were joined to give a continuous sequence spanning from framework region 1 to within the constant region using fastq-join (ea-utils), and default settings. Initial quality filtering was performed to remove any sequences containing unknown nucleotides, or with a Phred quality <30 over more than 15% of bases. Sequences were then submitted to IMGT/HighV-Quest (23) for annotation, and unproductive sequences (as defined by IMGT) removed. To account for differences in the number of resulting sequences in different samples, all samples were randomly subsampled without replacement using the sample function in R (24) to give 100,000 sequences per sample.

### Sequence-Level Annotation

Germline V and J gene usage, CDR3 AA sequence, number of V gene mutations from germline, and constant region nucleotide sequence was determined for each sequence by IMGT. Constant region sequences were then mapped to germline using Stampy (25) to determine isotype subclass, and number of nucleotide mismatches. Number of nucleotide mismatches in the constant region was used for error rate estimation, as this region will not be subject to somatic hypermutation. For each CDR3 AA sequence, the distance to its nearest neighbor in the same sample was determined. The nearest neighbor was defined as the sequence with the same CDR3 AA length and the fewest mismatches, with the nearest neighbor distance being the count of these mismatches.

### Cluster-Level Annotation

Clustering was performed using a previously described method (4), to form groups of sequences which are sufficiently similar that they are likely to be clonally related, or differ due to PCR and sequencing error. To be considered part of the same cluster, sequences were required to have the same length CDR3, the same V and J gene annotation, and a similar CDR3 AA sequence. Different thresholds for CDR3 similarity were trialed, ranging from one AA mismatch allowed per four AA’s (≥75% similarity) to one AA mismatch per 26 AA’s (≥96% similarity). D gene annotation was not considered, as this can not be carried out with a high degree of certainty (26), and the underlying D gene sequence is anyway included in the CDR3 AA sequence. Sequences from all samples were clustered together to allow easy comparison of clusters between participants and over time. The number of participants each cluster was present in was determined, along with specific comparisons of clusters present in different replicates. Each cluster was annotated with its dominant “cluster center” CDR3 AA sequence, the total number of unique sequences within it from a particular sample, in addition to the mean V gene mutation of these sequences, and their isotype subclass usage. Nearest neighbor distances were also calculated for cluster center sequences in the same way they were calculated for the individual sequences. Clusters were annotated for having potential binding specificity toward either influenza or tetanus toxoid (TT) antigens based on comparison to previously published sequences known to be specific for these antigens (27–34). The comparison of clusters to these known sequences was based on CDR3 AA sequence identity only, and was based on whether the known sequence would have fallen into that cluster during the clustering process (i.e., allowing one AA mismatch per 12 AAs).

### Repertoire-Level Annotation

The mean CDR3 AA length, and number of V gene mutations were calculated for each sample using all sequences in the repertoire. In addition, the relative proportion of the repertoire comprised by sequences using different V and J genes (and VJ combinations), and of different isotype subclasses was calculated for each sample.

Repertoire diversity was calculated using three different single diversity metrics, which have previously been applied to repertoire studies. The Shannon entropy index and Simpson’s concentration index are derived from ecology and take into account species richness and abundance in a single sample; for the purpose of studying the BCR repertoire, each cluster is considered a distinct species. The Shannon index gives more weight to rare species while the Simpson index gives more weight to abundant species. In addition, a clonality index derived from cryptanalysis (the study of text-based ciphers) was used which measures the probability that sequences selected from different PCR replicate samples belong to the same cluster (35). As well as these single diversity indices, Hill-based diversity profiles of each repertoire were generated using the method described by Greiff et al. (36). These profiles are based on a continuum of diversity measures with different weighting (alpha values) ascribed to the abundant and rare species. Alpha values between 0 and 10 were used with a step value of 0.5.

### Statistical Analysis and Graphing

Statistical analysis was conducted in R (24), using ggplot2 (37) for constructing graphs, gplots for constructing heatmaps (38), and Circos (39) used for constructing circular plots. Rarefaction analysis was conducted using the Vegan R package (40), with individual clusters representing species, and sampling done without replacement. Extrapolation of the rarefaction curves was conducted based on Chao’s estimates (41), using the iNEXT package in R (42) with a *q* value of 0. Simpson’s concentration, Shannon entropy, and diversity profiles were all calculated using the Vegan R package (40). Diversity profiles were compared based on Euclidean distance and clustered using the complete linkage algorithm with the hclust function in the Stats R package (24). Principal component analysis was conducted using the prcomp R function in the Stats R package (24). Capture-recapture analysis was used to estimate the effective repertoire size using the Chapman-Estimator formula, which has previously been applied to BCR repertoires (43). Genotyping was carried out using TIgGER (44).

## Results

### Sequencing Output and Clustering

**Figure 2:**
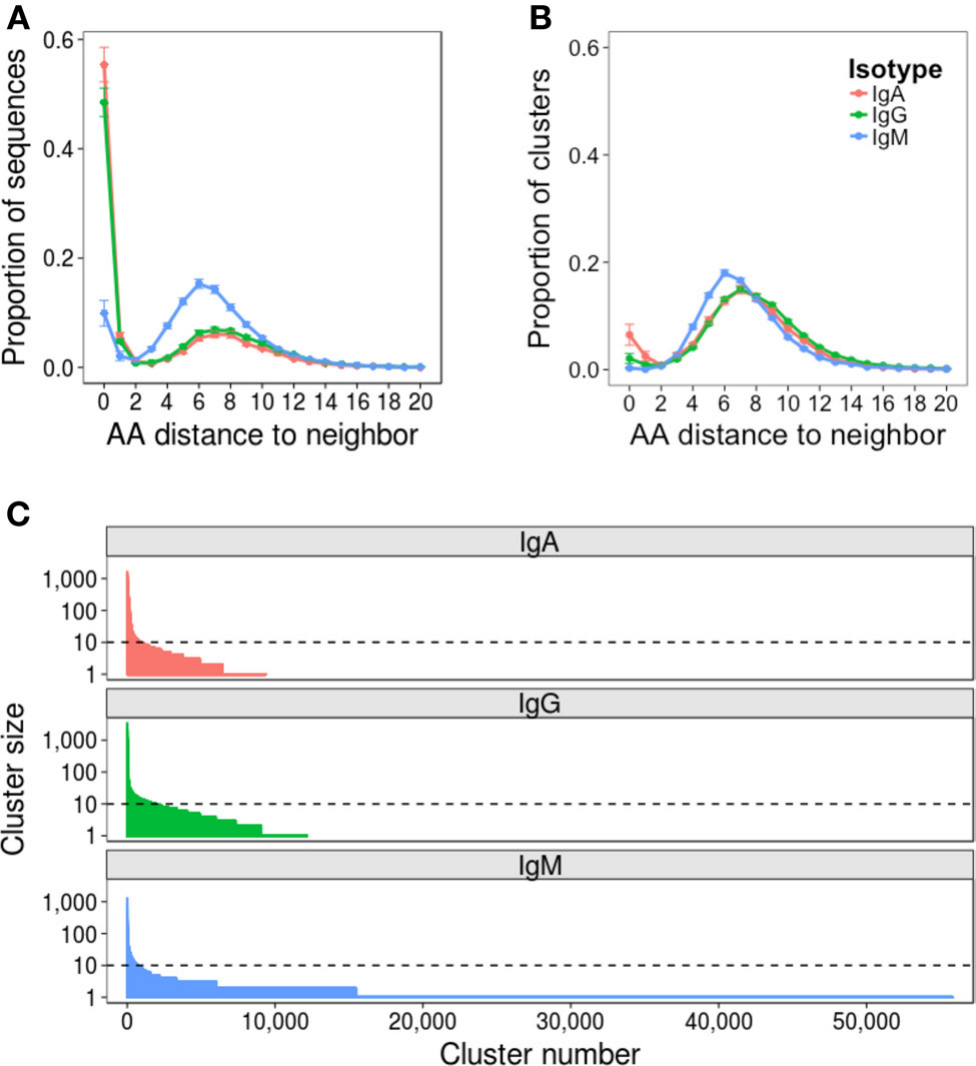
**Effect of clustering on the dataset**. Nearest neighbor distributions of sequences both before **(A)** and after **(B)** clustering. Before clustering, CDR3 AA sequences are used, and after clustering, the cluster center AA sequence is used. The nearest neighbor of each sequence in each dataset is determined by comparing it to every other sequence of the same length in the dataset to find the closest match – that is its nearest neighbor. The distance is then the number of AA difference between the sequence and its nearest neighbor. Distributions were calculated from samples from all 10 participants, and mean ± SEM values plotted. **(C)** Clusters are ordered according to size, and the size of each cluster plotted. Representative data from one sample is shown (participant AF01, day 0). Horizontal dotted line intersects the *y*-axis at 10 sequences (0.01% of the total sequenced repertoire), and represents the cutoff between rare and abundant clusters.

Repertoire data were successfully obtained for all 52 samples (Table S1 in Supplementary Material). The mean number of raw sequences per sample was 367,634 (216,878–1,516,275). Quality filtering removed on average 31% of raw sequences, leaving at least 100,000 sequences per sample for subsequent analysis. Error rate estimates differed depending on the isotype of the sequence, and were 0.0021, 0.0079, and 0.0019 errors per nucleotide for IgA, IgG, and IgM sequences, respectively (Figure S1 in Supplementary Material). To mitigate the effect of the error on subsequent analyses, data were clustered to group together closely related sequences. Analyzing the AA distance between CDR3 sequences prior to clustering revealed a bimodal distribution: the first peak of sequences had a close neighbor 0–2 AAs away, and the second peak of sequences had a more distant neighbor 3–15 AAs away (Figure 2A). The first peak was higher for IgA and IgG compared to IgM sequences, and the position of the second peak was shifted 1 AA toward the *y*-axis for the IgM sequences. As the first peak likely contains sequences whose nearest neighbor is either clonally related or differs due to error and the second peak likely contains sequences whose nearest neighbor arises from a distinct B cell clone, clustering should ensure that sequences with a neighbor in the first peak are clustered together, but sequences with a neighbor in the second peak are not. To find the clustering threshold that best achieved this, different thresholds allowing between one AA mismatch per every four AA’s to one AA mismatch per every 26 AA’s (i.e., from ≥75% to ≥96% similarity) in the CDR3 AA sequence were trialed. Above a threshold of one AA mismatch per 12 AA’s there is a failure to cluster some sequences differing by two AA’s (Figure S2A in Supplementary Material), and below a threshold of one AA mismatch per eight AA’s, there is a sharp drop in the number of clusters formed (Figure S2B in Supplementary Material), indicating that sequences from unrelated cells start to be grouped. A threshold of one AA mismatch per 12 AA’s was, therefore, chosen for the final analysis; however, it should be noted that using a threshold anywhere between one AA mismatch per eight AA’s and one AA mismatch per 12 AA’s gave negligible difference to the conclusions presented here.

Following final clustering with the threshold of one AA mismatch per 12 AA’s, the mean number of clusters differed for the different isotypes, and was 9,972 for IgA (1,658–22,940), 15,080 for IgG (1,599–25,000), and 53,150 for IgM (19,000–75,420) (Table S1 in Supplementary Material). Following clustering, the amplitude of the first peak of the nearest neighbor distribution of cluster center CDR3 AA sequences was greatly reduced for IgA and IgG sequences, and completely removed for IgM sequences (Figure 2B). Cluster sequences with a distance of 0 to their neighbor represent clusters with the same CDR3, but different V and/or J gene annotations. Although such sequences could be chimeras formed during the PCR reaction, they are not present in the IgM dataset, making this unlikely, and were, thus, retained for analysis; it has also previously been reported that such sequences can form during convergent evolution in response to antigen (12). The size distribution of clusters was highly uneven, with a small number of abundant clusters, and a large number of rare clusters (Figure 2C). Based on this, we defined clusters as abundant if they contained at least 10 sequences (i.e., comprised at least 0.01% of the total sequenced repertoire). Datasets contained mean 930 (518–1,537), 835 (195–1,983), and 642 (195–1,983) abundant clusters for IgA, IgG, and IgM, respectively.

### Quantitative Assessment of Sequencing Depth

**Figure 3:**
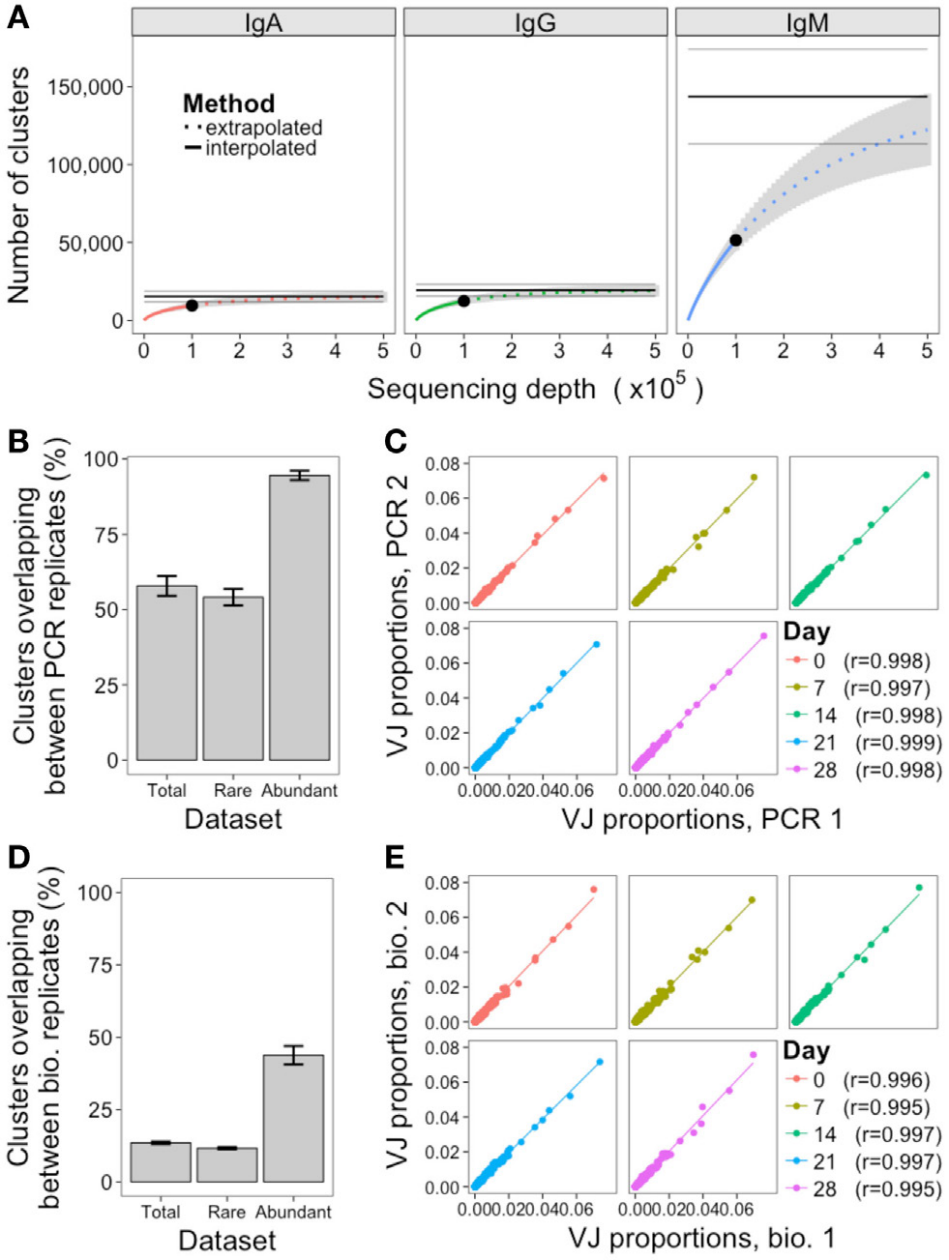
**Assessment of sequencing and sampling depth**. **(A)** Extrapolated rarefaction curves for clusters of each isotype. Rarefaction analysis (interpolated) was conducted by subsampling data without replacement at 1,000 sequence increments, and determining the number of clusters represented by these sequences. Cluster richness estimation of the sample (extrapolation) was based on Chao’s estimator formula, and conducted up to a sequencing depth of 500,000 sequences. The curve shows the number of clusters identified as a function of sampling depth – as the curves start to plateau it indicates that increased sampling depth will yield few additional clusters, and sampling depth is sufficient. Results were calculated from samples from all 10 participants, and mean ± SEM (gray curve) plotted. The three horizontal gray lines show the mean ± SEM for the Chao estimate of total clusters in the sample. **(B)** For the PCR replicates, the percent of clusters present in both replicates was determined, where percent = (*A*∩*B*/min(*A*,*B*)) × 100. This was determined for total clusters, rare clusters (<10 sequences), and abundant clusters (≥10 sequences) for all 10 participants, and mean ± SEM values plotted. **(C)** Correlation in proportion of the total repertoire comprised by each VJ gene combination in the two PCR replicates at each day. Each point represents the proportional representation of a particular VJ combination in the repertoire. *r*-Values represent Pearson’s correlation coefficients. **(D,E)** Same as **(B,C)**, but comparing the biological replicates.

Two methods were used to assess the adequacy of the sequencing depth used: rarefaction analysis, and comparison of the PCR replicates. Rarefaction is a technique used in ecology to estimate species richness; in the context of the BCR repertoire, each cluster is defined as a unique species. Random samples of increasing size were taken from the total dataset to determine the number of clusters that were represented at increasing sequencing depths, and a curve was drawn to show the number of clusters represented as a function of sequencing depth (Figure 3A). As the curve plateaus, it indicates that sequencing depth is sufficient, and only the very rare clusters remain to be identified. The curves for IgA and IgG do show the beginning of a plateau, so we can infer that a sequencing depth of 100,000 is sufficient to capture most of the abundant clusters for these samples. For IgM samples, which had a much larger number of rare clusters (Figure 2C), the curve does not begin to plateau, so we are unlikely to be capturing the full diversity of this population. If the curve does not plateau, it can be extrapolated to estimate where the plateau would occur, and give an estimate of the effective population size (a lower bound for the total number of clusters) of the population. However, these extrapolated values should be treated as rough values only, as they assume that all clusters will be present at the same frequency, and become unreliable once extrapolated to twice the interpolated number (41). Using the extrapolated curves to estimate total number of clusters for the different samples gives a mean value of 15,414 total clusters for IgA, 19,488 for IgG, and 143,731 for IgM, indicating that our sequencing depth captures ~63, 64, and 36% of all IgA, IgG, and IgM clusters, respectively. In order to capture 90% of clusters contained in a sample of 500,000 B cells, it would, therefore, be necessary to obtain ~280,000 sequences for IgA, 260,000 for IgG, and 620,000 for IgM.

PCR replicates were only conducted for IgG samples, so comparison of these can only be used to assess sequencing depth for this isotype. Although simply re-sequencing the same library can also be used to assess sequencing depth, conducting the PCR again prior to sequencing is a more stringent measure that will also take into account differences in amplification efficiency of the template cDNA. Across the five samples where PCR replicates were available, the mean overlap of clusters present in both PCR replicates was 58%, but this increased to 95% when just considering the abundant clusters (Figure 3B; Figure S3 in Supplementary Material). In addition to identification of specific sequences, many repertoire studies also assess relative proportions of different VJ combinations. To determine the reproducibility of this, the proportional representation of each VJ clone in the repertoire was determined for each sample, and correlated. Strong correlations were seen, with Pearson’s *r* > 0.997 for all PCR replicate samples (Figure 3C).

### Quantitative Assessment of Sampling Depth

To assess the adequacy of sampling 500,000 B cells for assessment of the total BCR repertoire, we used the biological replicates to estimate a lower bound for the total IgG BCR cluster repertoire size using capture-recapture analysis. Across the five samples where biological replicates were available, the mean IgG effective repertoire size was estimated as 142,576 unique clusters; as we obtained a mean of 15,128 total IgG clusters from these samples, we estimate that ~11% of the total repertoire was sampled at each timepoint (Table 1). Taking just the abundant clusters, the mean estimated BCR repertoire size of abundant clusters was 4,644, indicating that we sample ~25% of the abundant repertoire at each timepoint. Estimates of repertoire size varied on the different days of sampling, and this variation in size estimates was more pronounced for the abundant repertoire (up to 8.9× size difference between days) compared to the total repertoire (up to 1.5× size difference between days).

**Table 1:** **Number of total clusters, and abundant clusters for the IgG 1 sample at each timepoint from participant AF01, size estimates of the total IgG and abundant IgG cluster repertoire based on capture-recapture analysis of biological replicates, and the percent of the total repertoire, and abundant repertoire that we sequenced**.

Day	Total clusters sampled	Abundant clusters sampled	Total clusters size estimate	Abundant clusters size estimate	Total clusters sampled (%)	Abundant clusters sampled (%)
0	12,152	1,919	132,841	10,426	9.1	18.4
7	13,186	367	108,253	1,168	12.2	31.4
14	16,907	1,137	162,868	5,368	10.4	21.2
21	17,930	861	158,311	3,013	11.3	28.6
28	15,463	775	150,605	3,245	10.3	23.9
Mean	15,128	1,012	142,576	4,644	10.7	24.7

As with the PCR replicates, the overlap in clusters present in both of the biological replicates was also calculated (Figure 3D; Figure S3 in Supplementary Material). The mean overlap of total clusters present in both biological replicates was 14%, although this increased to 44% when considering the abundant clusters only. The reproducibility of VJ usage frequency remained strongly correlated between the biological replicates, with Pearson’s *r* > 0.995 for all samples (Figure 3E).

### Fluctuations in the Repertoire over Time

**Figure 4:**
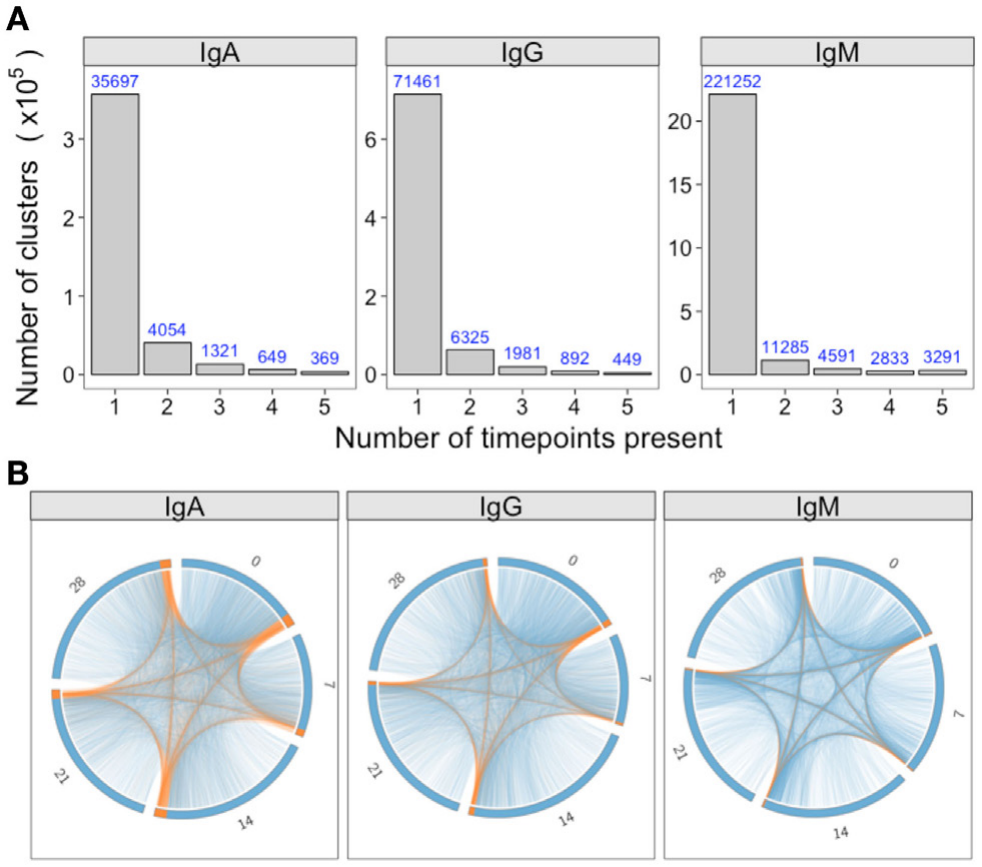
**Persistence of clusters over time**. **(A)** For samples of each isotype from participant AF01, the total number of clusters across all timepoints was determined, and the number of these present at different number of timepoints calculated. **(B)** Using the same data as A, circos plots were constructed to show the clusters present on different days. Each arc represents a different day, and clusters are ordered clockwise by abundance, with the abundant clusters colored orange, and the rare clusters colored blue. Lines join clusters present at more than one timepoint.

For a single participant, individual clusters were tracked across the samples collected at different times to see if they could be detected on multiple days. Although most clusters were present on just a single day, 5, 14, and 7% were detected on more than 1 day for IgA, IgG, and IgM, respectively (Figure 4A). In addition, there were a small number of clusters detected at all timepoints. Circos plots were constructed to show the relationship between the clusters present on different days, and show that it is primarily the abundant clusters that are present on more than 1 day (Figure 4B).

**Figure 5:**
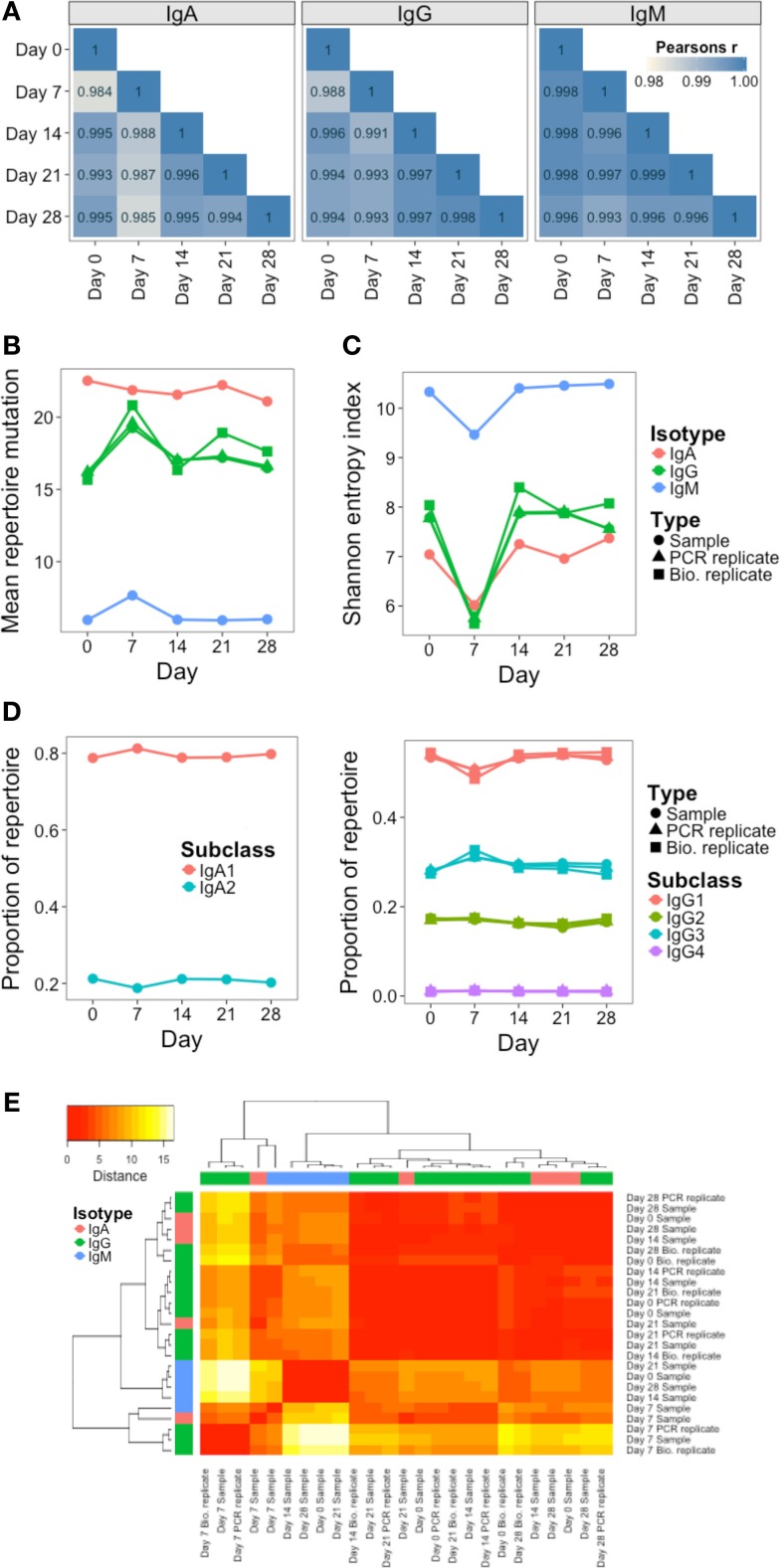
**Persistence of global repertoire properties over time**. **(A)** Correlation in relative usage proportion of each VJ gene combination from samples on different days from participant AF01. Stronger correlations are in darker blue. **(B)** Change in the mean number of V gene mutations of sequences in the repertoire over time. **(C)** Change in repertoire diversity over time, calculated using the Shannon entropy index, with each cluster represented as a distinct species. **(D)** Change in proportion of the repertoire comprised by sequences of the different IgA and IgG subclasses over time. **(E)** Euclidean distances between the diversity profiles of each sample were calculated, used for hierarchical clustering of the samples, and visualized as a heatmap. The color of the heatmap indicates the similarity in the profile between two samples (red = high similarity, white = low similarity). The color bars indicate the isotype of each sample.

In addition to monitoring individual clusters, global repertoire metrics of VJ segment usage (Figure 5A), mutation (Figure 5B), diversity (Figure 5C), and isotype subclass (Figure 5D) were determined at each day. Different single measurements for diversity were used, but they all gave the same trend (Figure S4 in Supplementary Material). Although most clusters do not persist over time, VJ segment usage frequency remained highly correlated between all days. Although the other repertoire metrics were highly conserved between both the PCR and biological replicates, on day 7, there was a large fluctuation in the repertoire, causing an increase in mutation and a decrease in diversity (most prominently in the IgG datasets), and a relative decrease in IgG1, and increase in IgG3 usage. Although there were actually fewer abundant clusters on day 7 than on the other days, the size of these abundant clusters tended to be greater (Figure S5 in Supplementary Material), suggestive of clonal expansions occurring at this timepoint. The use of diversity profiles has recently been reported as a more accurate method for determining immunological status from BCR data than the use of single diversity measures (36), and these results are shown in Figure 5E. The difference between the diversity profiles of different samples can then be calculated, and used for hierarchical clustering of the samples based on diversity. This revealed that all day 7 samples clustered together regardless of isotype. All IgM samples also clustered together, but the distinction between IgA and IgG samples was not as clear.

### Inter-Individual Variation in the Repertoire

**Figure 6:**
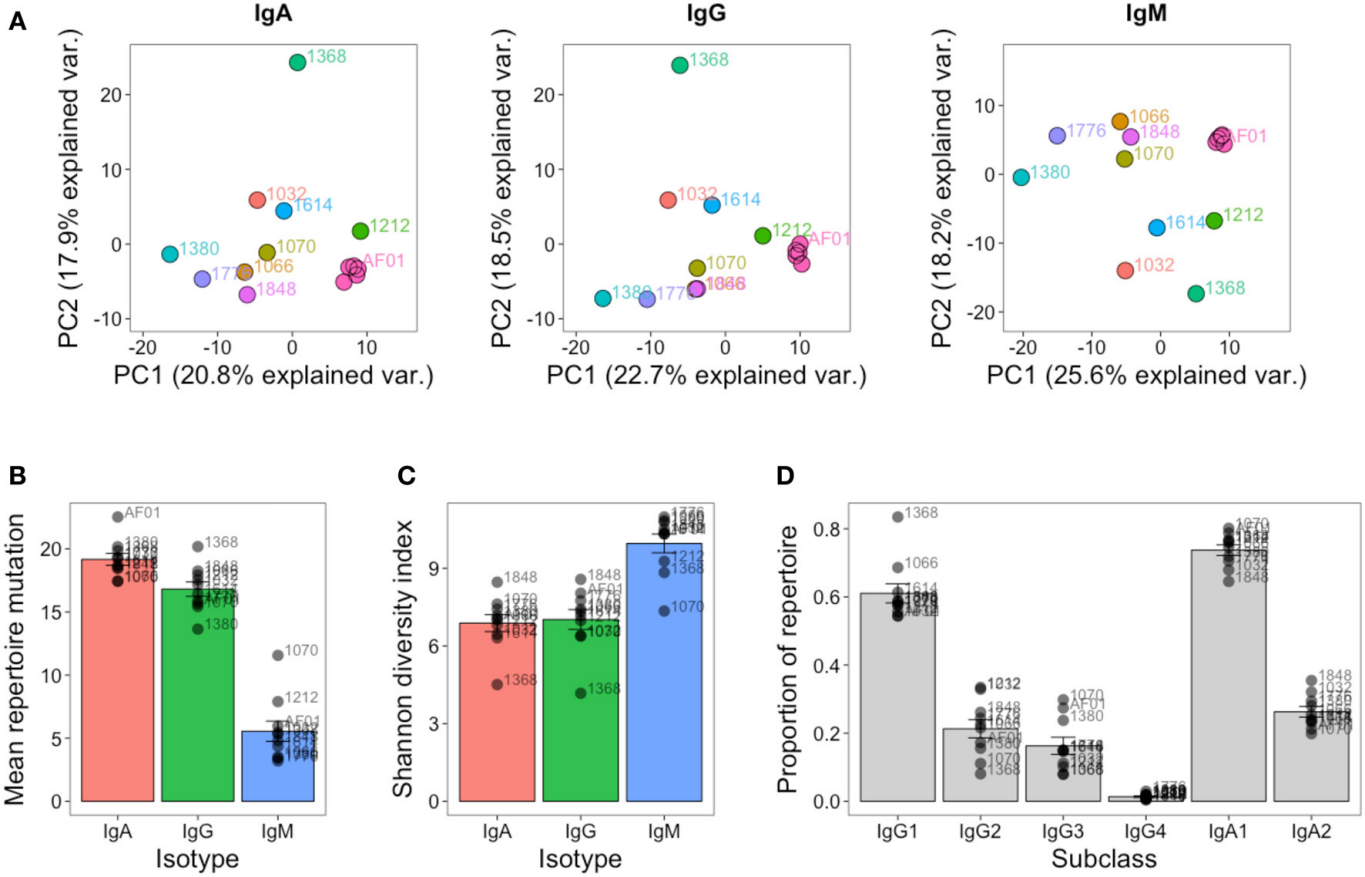
**Inter-individual variation in global repertoire properties**. **(A)** Principal component analysis of VJ segment usage in each participant. Five samples from participant AF01 are included, each corresponding to a sample from a different day. Differences in mean number of V gene mutations of sequences in the repertoire **(B)**, repertoire diversity (calculated using the Shannon diversity index) **(C)**, and proportion of the repertoire comprised by sequences of each IgG and IgA subclass **(D)** in each participant. For **(B–D)**, bars show mean values ± SEM.

As well as the single highly sampled participant, nine additional participants were sampled at a single timepoint to give insight into inter-individual variation in the repertoire. Although at the cluster level, there was very little overlap between the repertoires of different participants (Figure S3 in Supplementary Material), the repertoires of different participants were more comparable at the level of the general repertoire properties. Different V and J gene segments are not used in even proportions, and these biases in gene use are conserved between different participants (Figure S6 in Supplementary Material). To more specifically assess any differences in VJ usage between participants, principle component analysis was used. This is a dimensional reduction technique that takes into account independent correlations in usage frequencies between variables (VJ combinations) to give components that can explain the largest proportion of the total variability in the data. The first two principle components account for the largest proportion of variability, and in the case of VJ usage, account for ~40% of the variability. Plotting the samples according to the first two principle components shows that the samples from different participants cluster apart from each other compared to the samples on different days from participant AF01 which cluster closely together, indicating that VJ usage frequency is able to uniquely identify the different participants, and remains steady in participant AF01 despite apparent immune activation (Figure 6A). Furthermore, the V gene genotype was inferred for each participant, indicating that each participant had a unique V gene genotype (Table S2 in Supplementary Material). Ten putative novel alleles were also found, that were not contained within the IMGT database; two of these have previously been reported, giving good evidence for them being real alleles (44).

The global repertoire properties of mutation (Figure 6B), diversity (Figure 6C), and isotype subclass usage (Figure 6D) were also determined for each participant. There was considerable variation in each of these properties between the participants. Participant 1368 appeared to consistently be an outlier in nearly all measures for all isotypes, and notably for IgG had a highly mutated repertoire with low diversity, which is indicative of clonal expansions. The study was not powered to detect age-related differences in the repertoire, but we had an even mix of participants between the ages of 22 and 59. We correlated each of the global repertoire properties with age, and found a significant decrease in IgG2 usage with age, giving a relative increase in IgG1 and IgG3 use (Figure S7 in Supplementary Material). Although not significant, other global repertoire metrics also appeared to change with age (increase in mutation, and decrease in diversity with age), and warrant exploration with appropriately powered studies.

### The Public Repertoire

**Figure 7:**
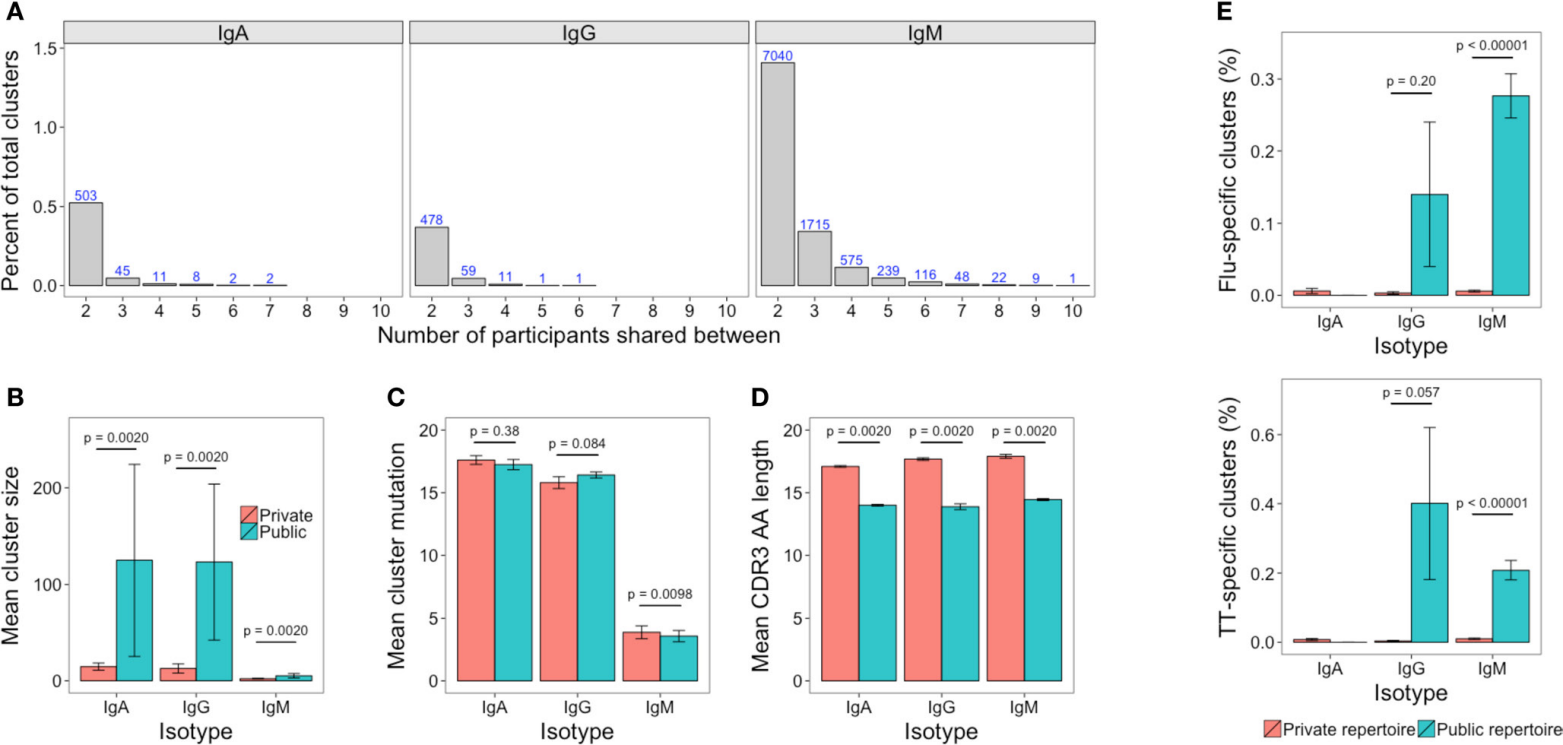
**The public repertoire**. **(A)** The percent of total clusters that are present in different numbers of participants, where percent = (*A*∩*B*/sum(*A*,*B*)) × 100. The blue number above each bar shows the absolute number that is shared. **(B–D)** Mean cluster size, mutation, and CDR3 AA sequence length in the clusters that are unique to a participant (private repertoire) compared to those that are present in at least one other participant (public repertoire). **(E)** Percent of clusters in the private and public repertoire that are annotated as having specificity toward either TT or Influenza antigens. For **(B–E)**, mean values ± SEM are shown for the 10 participants. Comparisons performed using the paired Mann-Whitney U test.

The public repertoire is defined as set of clusters within the repertoire that are common to multiple participants, whereas the private repertoire is the set of clusters that are unique to a particular participant. In an unstimulated setting, the public repertoire comprises a very minor part of the total repertoire, but is greater for IgM than IgG or IgA (1.4, 0.3, and 0.5% of total clusters, respectively). Considering the number of clusters present in different numbers of participants, there was a sharp reduction when considering clusters shared by increasing numbers of participants, and there was only a single cluster (IgM) that was present in all participants (Figure 7A). Compared to the private repertoire, the public repertoire comprised larger clusters that had shorter CDR3’s (Figures 7B,D). For IgG, the public clusters were more mutated than the private clusters, but for IgM, the public clusters were actually less mutated than the private clusters, with no significant difference for IgA (Figure 7C).

Comparing previously described sequences specific for TT or influenza to out dataset revealed 50 TT-specific (of 1,093), and 19 influenza-specific (of 321) sequences that mapped to clusters in our dataset (Table S3 in Supplementary Material). These clusters were, therefore, labeled as potentially containing sequences with specificity toward TT and influenza, respectively. The potential TT and influenza-specific clusters were present in samples of at least one isotype from each participant, but only in small numbers. The mean number of potential TT-specific and influenza-specific clusters was 1.1 and 0.5, respectively for IgA, 1.0 and 0.6 for IgG, and 10.5 and 9.4 for IgM. Despite the low numbers, where they were present, potential TT and influenza-specific clusters comprised a greater percentage of the public compared to the private repertoire (Figure 7E).

## Discussion

By collecting both repeat samples from a single participant as well as samples from multiple different participants, we have been able to perform in-depth assessment of both the within-individual, and inter-individual variation in the BCR repertoire. We show high reproducibility in the methods used to sample the BCR repertoire, and although not exhaustively sampling the entire repertoire at the individual B cell clone level, calculate comparable global repertoire metrics, and routinely detect abundant clusters in repeat samples. In the absence of an immune stimulus given during the study, there was considerable variation in the BCR repertoire over time in a single individual, highlighting that it is a highly dynamic system that is constantly subject to immune stimulus and selective pressures. Nevertheless, there are certain features that remain steady, which enable unique identification of different individuals, and may be a contributing factor to genetic causes of variation in the immune response. Finally, we show that there is a small public repertoire, and that this has a distinct structure, and appears enriched for specificity toward commonly encountered antigens.

Although there is no standardized BCR repertoire sequencing protocol, different protocols give comparable results (45), so the data generated here are still informative for laboratories using alternate protocols. We favored use of a simple and cost-effective protocol, so that it could potentially be used on a large scale in the context of clinical trials, or for routine diagnostic tests. As input, we used 500,000 B cells – this number can normally be obtained from 2 to 8 ml of peripheral blood, so is a feasible quantity for routine sampling, including samples obtained from the youngest infants. To account for sequencing and PCR error in the dataset, a clustering-based approach was used that groups together related sequences. This approach is intended to just group together sequences arising from the same cell that only differ due to sequencing or PCR error; however, it is likely that sequences arising from clonally related cells, or cells with similar specificities will also be grouped together. Indeed, other laboratories have used similar approaches to identify what they term to be clones, or clonotypes (9, 46), but this could be misleading, as B cells from distinct clonal origins could converge toward a similar sequence (12). For this reason, we instead consider the clusters to be groups of sequences that likely have a similar antigenic specificity. Using clustering for error correction is cheaper than using unique molecular identifier tags (43), which require increased depth of sequencing and may have other associated errors (47). We detected a small number of clusters that had identical CDR3 AA cluster center sequences, which were combined with different V and J gene segments. Such sequences could be PCR chimeras, or form during convergent evolution of different B cells toward the same antigen (12). We favor the latter explanation, as these clusters were only present in the IgG and IgA datasets, and not the IgM dataset, which is mainly comprised of naïve B cells that have not undergone any selection. There was a large size distribution of clusters ranging from those just containing a single sequence (0.001% of the total sequenced repertoire) to 19,662 sequences (nearly 20% of the repertoire). This could in part be due to different PCR amplification efficiencies of different templates, the presence of different numbers of clonally related cells from which sequences are being grouped together, or different RNA quantities in different B cell subsets. Larger clusters are, therefore, more likely to represent plasma cells (which have a large amount of RNA), or proliferating cells (of which there will be multiple similar cells).

Obtaining both PCR and biological replicates allowed us to assess the adequacy of our sequencing depth for representing the true diversity of the 500,000 B cells in the sample, and the adequacy of our sampling depth for representing the true diversity of total B cells in a human. Sequencing depth and sampling depth are related in that there is no point collecting a large sample if sequencing depth is insufficient to capture the diversity of the sample. Under-sequencing results in a loss of information, whilst over-sequencing represents an unnecessary cost and increases the number of erroneous sequences in the dataset. Rarefaction analysis indicated that whilst we are not capturing the entire diversity of the 500,000 B cells, we are reliably capturing the most abundant clusters. So, for most applications, our sequencing depth should be sufficient, but for rare cluster identification, a greater sequencing depth would be recommended, especially for the IgM population, which forms the greatest proportion of the total B cells. For isotype-specific BCR repertoire sequencing from a pool of total B cells, in order to reliably sequence 90% of cells in the sample, we recommend aiming for at least a 0.5:1 ratio of sequences to cells for IgA and IgG, and a 1:1 ratio of sequences to cells for IgM populations. Using the biological replicates, the estimates of total IgG repertoire size we determined were steady over time, and in line with previous studies (43, 48). Considering just the abundant clusters, estimates varied by nearly a factor of 10 at the different times, indicating that the repertoire of these clusters is more likely to be affected by immune fluctuations. It is worth emphasizing though, that our repertoire size estimates should be considered a lower bound for the total number of circulating clones. Sequences from similar clones may be clustered together, and the data violate the capture-recapture analysis assumption of equal abundance of all clusters, which will cause the size estimates to be deflated.

At day 7, the dramatic changes in mean mutation levels (increase), diversity (decrease), and isotype subclass usage are indicative of potential B cell activation (4). We, therefore, hypothesize that these changes were due to a subclinical immune activation in this participant at day 7. In addition, the abundant repertoire had the smallest estimated repertoire size at day 7, potentially due to a restriction toward more limited antigenic specificity. Despite their reduction in number, the abundant clusters were large and mutated, so likely composed of rapidly proliferating B cells (49). The day 7 suspected immune stimulus highlights how dynamic the repertoire is over time, so for studies of the BCR repertoire following immune stimulation, it must, therefore, be considered that there could be natural stimulations of the repertoire that could affect the results. Further evidence for this comes from studying the differences between participants, where one had high mutation, and low repertoire diversity, indicative of immune stimulation in this participant as well. A larger study of more participants at multiple timepoints is necessary to determine exactly how common such immune stimulations are in normal healthy individuals, and how long they tend to last. In addition, further exploration of the optimal statistical approach for measuring BCR repertoire diversity is required (50). We used three different single diversity measures that have previously been applied to BCR repertoire studies (8, 35). These all gave similar results, and were adequate for detecting the change at day 7. However, it has been reported that the use of different single diversity measures could give different results when comparing BCR repertoires, depending on the weight they give to abundant vs. rare species (36). For this reason, we also used the diversity profile method for comparing repertoires as suggested by Greiff et al. (36), which was also able to distinguish the day 7 samples from the other days.

Despite the day 7 changes in the repertoire, the proportion of usage of different VJ gene combinations remained steady over time in a single individual, compared to the relatively large differences between individuals. As VJ usage, therefore, appears unique to an individual, and certain V genes are preferentially used in a protective response to certain antigens (51, 52), it could potentially be a cause of variation in disease and vaccine responses between individuals. As well as VJ usage frequency, V gene genotype is also unique for each individual in this study, and as certain V gene alleles may have different binding abilities to certain antigens (53), this may also affect disease and vaccine response. Although isotype subclass usage, mutation and diversity are more affected by immune stimulation, they also display considerable inter-individual variation. We hypothesized that one cause of this could be the different ages of the individuals in the study, as it has previously been observed that the repertoire in more elderly participants is less diverse and more mutated (9, 35). We also saw a decrease in diversity, and increase in mutation with age, although this was not a statistically significant finding. Most striking, however, was a significant decrease in IgG2 levels with age, coinciding with an increase in IgG1 and IgG3. Such an observation is of potential importance, as the different IgG subclasses have different activities in different antigenic contexts (54), and change in their relative abundance could be a contributing factor to immunosenescence (55). For example, IgG2 is important for mounting immune responses against polysaccharide-encapsulated pathogens (such as *Neisseria meningitides* and *Streptococcus pneumonia*) (56). Such responses are reduced in older individuals (57), so this could be due to decreased IgG2 levels.

It appears that when different individuals are exposed to a common antigenic stimulus, there is a degree of similarity in the response (a public repertoire) at the BCR sequence level, and that this could be used to identify antigen-specific BCR sequences (4, 11, 17, 21). However, here we also observe the presence of a public repertoire in the absence of any common immune stimulation. The presence of such a public repertoire could have three possible causes: laboratory contamination of different samples, random overlap by chance, or historical common antigenic stimuli. Laboratory work was conducted under stringent conditions to minimize cross-sample contamination, and there are no clusters shared across all samples, making this an unlikely contributor to the public repertoire. If sharing was due to chance, it is expected that the public and private repertoires would have similar properties, but this is not the case. The public IgG repertoire comprises larger, more mutated clusters, with shorter CDR3s than the private repertoire; this is consistent with these clusters arising from more differentiated B cell subsets (4). In addition, considering the presumed antigenic specificity of the clusters, a greater proportion of the public repertoire comprised presumed TT or influenza-specific clusters compared to the private repertoire. These are antigens to which all participants in the study are likely to have been exposed through either vaccination or infection, and thus provide support for using public repertoire analysis for identification of antigen-specific clusters following common antigen stimulation. However, it must be considered that this technique could then also enrich for sequences specific to antigens that are commonly encountered by the population. The public repertoire in the IgM dataset is approximately three times larger than that of the IgG or IgA datasets. This may be due to the presence of natural IgM antibodies, which target conserved microbial determinants and autoantigens (58); the most abundant of these is anti-gal, which constitutes ~1% of all human antibodies (59). This could explain why the public repertoire is less mutated than the private repertoire for IgM sequences, while the converse is true for IgG sequences. Unfortunately, to our knowledge there are currently no large sequence datasets of natural antibodies available to search in our dataset to confirm this hypothesis.

To summarize, we present a robust BCR repertoire sequencing method, and demonstrate that a sample of 500,000 B cells, and sequencing depth of 100,000 sequences per B cell isotype should be sufficient for most applications. The BCR repertoire varies substantially within a single individual over time, and between multiple different individuals. Assessing the complete range of such variation would benefit from further characterization in larger study cohorts. Nevertheless, we show certain conserved features of the repertoire within individuals that could be predictive of immune function. Finally, we investigated the public repertoire, and show that this is likely to be enriched for sequences with specificity toward antigens commonly encountered by the population.

## Conflict of Interest Statement

The authors declare that the research was conducted in the absence of any commercial or financial relationships that could be construed as a potential conflict of interest.

## Supplementary Material

The Supplementary Material for this article can be found online at http://journal.frontiersin.org/article/10.3389/fimmu.2015.00531

## References

[1] Glanville J Zhai W Berka J Telman D Huerta G Mehta GR Precise determination of the diversity of a combinatorial antibody library gives insight into the human immunoglobulin repertoire. Proc Natl Acad Sci U S A (2009) 106:20216–21. 10.1073/pnas.0909775106 19875695 PMC2787155

[2] Tonegawa S. Somatic generation of antibody diversity. Nature (1983) 302:575–81. 10.1038/302575a0 6300689

[3] Victora GD Nussenzweig MC. Germinal centers. Annu Rev Immunol (2012) 30:429–57. 10.1146/annurev-immunol-020711-075032 22224772

[4] Galson JD Clutterbuck EA Trück J Ramasamy MN Münz M Fowler A BCR repertoire sequencing: different patterns of B-cell activation after two meningococcal vaccines. Immunol Cell Biol (2015). 10.1038/icb.2015.57 25976772 PMC4551417

[5] Mroczek ES Ippolito GC Rogosch T Hoi KH Hwangpo TA Brand MG Differences in the composition of the human antibody repertoire by B cell subsets in the blood. Front Immunol (2014) 5:96. 10.3389/fimmu.2014.00096 24678310 PMC3958703

[6] DeKosky BJ Kojima T Rodin A Charab W Ippolito GC Ellington AD In-depth determination and analysis of the human paired heavy- and light-chain antibody repertoire. Nat Med (2015) 21:86–91. 10.1038/nm.3743 25501908

[7] Xu JL Davis MM. Diversity in the CDR3 region of V(H) is sufficient for most antibody specificities. Immunity (2000) 13:37–45. 10.1016/S1074-7613(00)00006-6 10933393

[8] Rechavi E Lev A Lee YN Simon AJ Yinon Y Lipitz S Timely and spatially regulated maturation of B and T cell repertoire during human fetal development. Sci Transl Med (2015) 7:1–11. 10.1126/scitranslmed.aaa0072 25717098

[9] Jiang N He J Weinstein JA Penland L Sasaki S He X-S Lineage structure of the human antibody repertoire in response to influenza vaccination. Sci Transl Med (2013) 5:171ra19. 10.1126/scitranslmed.3004794 23390249 PMC3699344

[10] Wang C Liu Y Cavanagh MM Le Saux S Qi Q Roskin KM B-cell repertoire responses to varicella-zoster vaccination in human identical twins. Proc Natl Acad Sci U S A (2014) 112:500–5. 10.1073/pnas.1415875112 25535378 PMC4299233

[11] Jackson KJL Liu Y Roskin KM Glanville J Hoh RA Seo K Human responses to influenza vaccination show seroconversion signatures and convergent antibody rearrangements. Cell Host Microbe (2014) 16:105–14. 10.1016/j.chom.2014.05.013 24981332 PMC4158033

[12] Trück J Ramasamy MN Galson JD Rance R Parkhill J Lunter G Identification of antigen-specific B cell receptor sequences using public repertoire analysis. J Immunol (2014) 194:252–61. 10.4049/jimmunol.1401405 25392534 PMC4272858

[13] Wu Y-CB Kipling D Dunn-Walters DK. Age-related changes in human peripheral blood IGH repertoire following vaccination. Front Immunol (2012) 3:193. 10.3389/fimmu.2012.00193 22787463 PMC3391689

[14] Palanichamy A Apeltsin L Kuo TC Sirota M Wang S Pitts SJ Immunoglobulin class-switched B cells form an active immune axis between CNS and periphery in multiple sclerosis. Sci Transl Med (2014) 6:ra106–248. 10.1126/scitranslmed.3008930 25100740 PMC4176763

[15] Boyd SD Marshall EL Merker JD Maniar JM Zhang LN Sahaf B Measurement and clinical monitoring of human lymphocyte clonality by massively parallel V-D-J pyrosequencing. Sci Transl Med (2009) 1:12ra23. 10.1126/scitranslmed.3000540 20161664 PMC2819115

[16] Logan AC Zhang B Narasimhan B Carlton V Zheng J Moorhead M Minimal residual disease quantification using consensus primers and high-throughput IGH sequencing predicts post-transplant relapse in chronic lymphocytic leukemia. Leukemia (2013) 27:1659–65. 10.1038/leu.2013.52 23419792 PMC3740398

[17] Parameswaran P Liu Y Roskin KM Jackson KKL Dixit VP Lee J-Y Convergent antibody signatures in human dengue. Cell Host Microbe (2013) 13:691–700. 10.1016/j.chom.2013.05.008 23768493 PMC4136508

[18] Reddy ST Ge X Miklos AE Hughes RA Kang SH Hoi KH Monoclonal antibodies isolated without screening by analyzing the variable-gene repertoire of plasma cells. Nat Biotechnol (2010) 28:965–9. 10.1038/nbt.1673 20802495

[19] Liberman G Benichou J Tsaban L Glanville J Louzoun Y. Multi step selection in Ig H chains is initially focused on CDR3 and then on other CDR regions. Front Immunol (2013) 4:274. 10.3389/fimmu.2013.00274 24062742 PMC3775539

[20] Greiff V Menzel U Haessler U Cook SC Friedensohn S Khan TA Quantitative assessment of the robustness of next-generation sequencing of antibody variable gene repertoires from immunized mice. BMC Immunol (2014) 15:40. 10.1186/s12865-014-0040-5 25318652 PMC4233042

[21] Galson JD Pollard AJ Trück J Kelly DF. Studying the antibody repertoire after vaccination: practical applications. Trends Immunol (2014) 35:319–31. 10.1016/j.it.2014.04.005 24856924

[22] Wu Y-C Kipling D Leong HS Martin V Ademokun AA Dunn-Walters DK. High-throughput immunoglobulin repertoire analysis distinguishes between human IgM memory and switched memory B-cell populations. Blood (2010) 116:1070–8. 10.1182/blood-2010-03-275859 20457872 PMC2938129

[23] Brochet X Lefranc M-P Giudicelli V. IMGT/V-QUEST: the highly customized and integrated system for IG and TR standardized V-J and V-D-J sequence analysis. Nucleic Acids Res (2008) 36:W503–8. 10.1093/nar/gkn316 18503082 PMC2447746

[24] Team RDC. R: A Language and Environment for Statistical Computing. Vienna: R Found. Stat. Comput. (2008). Available from: http://www.r-project.org

[25] Lunter G Goodson M. Stampy: a statistical algorithm for sensitive and fast mapping of illumina sequence reads. Genome Res (2011) 21:936–9. 10.1101/gr.111120.110 20980556 PMC3106326

[26] Jackson KJL Boyd S Gaëta BA Collins AM. Benchmarking the performance of human antibody gene alignment utilities using a 454 sequence dataset. Bioinformatics (2010) 26:3129–30. 10.1093/bioinformatics/btq604 21036814

[27] Dekosky BJ Ippolito GC Deschner RP Lavinder JJ Wine Y Rawlings BM High-throughput sequencing of the paired human immunoglobulin heavy and light chain repertoire. Nat Biotechnol (2013) 31:166–9. 10.1038/nbt.2492 23334449 PMC3910347

[28] Frölich D Giesecke C Mei H. Secondary immunization generates clonally related antigen-specific plasma cells and memory B cells. J Immunol (2010) 185:3103–10. 10.4049/jimmunol.1000911 20693426

[29] Poulsen TR Jensen A Haurum JS Andersen PS. Limits for antibody affinity maturation and repertoire diversification in hypervaccinated humans. J Immunol (2011) 187:4229–35. 10.4049/jimmunol.1000928 21930965

[30] Poulsen TR Meijer P-J Jensen A Nielsen LS Andersen PS. Kinetic, affinity, and diversity limits of human polyclonal antibody responses against tetanus toxoid. J Immunol (2007) 179:3841–50. 10.4049/jimmunol.179.6.3841 17785821

[31] Krause JC Tsibane T Tumpey TM Huffman CJ Albrecht R Blum DL Human monoclonal antibodies to pandemic 1957 H2N2 and pandemic 1968 H3N2 influenza viruses. J Virol (2012) 86:6334–40. 10.1128/JVI.07158-11 22457520 PMC3372199

[32] Ohshima N Kubota-Koketsu R Iba Y Okuno Y Kurosawa Y. Two types of antibodies are induced by vaccination with A/California/2009 pdm virus: binding near the sialic acid-binding pocket and neutralizing both H1N1 and H5N1 viruses. PLoS One (2014) 9:e87305. 10.1371/journal.pone.0087305 24505283 PMC3914828

[33] Pappas L Foglierini M Piccoli L Kallewaard NL Turrini F Silacci C Rapid development of broadly influenza neutralizing antibodies through redundant mutations. Nature (2014) 516:418–22. 10.1038/nature13764 25296253

[34] Thomson CA Wang Y Jackson LM Olson M Wang W Liavonchanka A Pandemic H1N1 influenza infection and vaccination in humans induces cross-protective antibodies that target the hemagglutinin stem. Front Immunol (2012) 3:87. 10.3389/fimmu.2012.00087 22586427 PMC3347682

[35] Wang C Liu Y Xu LT Jackson KJL Roskin KM Pham TD Effects of aging, *Cytomegalovirus* infection, and EBV infection on human B cell repertoires. J Immunol (2013) 192:603–11. 10.4049/jimmunol.1301384 24337376 PMC3947124

[36] Greiff V Bhat P Cook SC Menzel U Kang W Reddy ST. A bioinformatic framework for immune repertoire diversity profiling enables detection of immunological status. Genome Med (2015) 7:3–5. 10.1186/s13073-015-0169-8 26140055 PMC4489130

[37] Wickham H ggplot2: Elegant Graphics for Data Analysis. 1st ed New York: Springer (2009).

[38] Warnes GR Bolker B Bonebakker L Gentleman R Huber W Liaw A gplots: Various R Programming Tools for Plotting Data, R Package. Version 2.17.0 (2015).

[39] Krzywinski M Schein J Birol I Connors J Gascoyne R Horsman D Circos: an information aesthetic for comparative genomics. Genome Res (2009) 19:1639–45. 10.1101/gr.092759.109 19541911 PMC2752132

[40] Oksanen J Blanchet FG Kindt R Legendre P Minchin PR O’Hara RB vegan: Community Ecology Package, R Package. Version 2.2.1 (2015).

[41] Chao A Gotelli NJ Hsieh TC Sander EL Ma KH Colwell RK Rarefaction and extrapolation with hill numbers: a framework for sampling and estimation in species diversity studies. Ecol Monogr (2014) 84:45–67. 10.1890/13-0133.1

[42] Hsieh TC Ma KH Chao A iNEXT Online: Interpolation and Extrapolation, R Package. Version 1.0 (2013).

[43] Vollmers C Sit R Weinstein JA Dekker CL Quake SR. Genetic measurement of memory B-cell recall using antibody repertoire sequencing. Proc Natl Acad Sci U S A (2013) 110:13463–8. 10.1073/pnas.1312146110 23898164 PMC3746854

[44] Gadala-Maria D Yaari G Uduman M Kleinstein SH. Automated analysis of high-throughput B-cell sequencing data reveals a high frequency of novel immunoglobulin V gene segment alleles. Proc Natl Acad Sci U S A (2015) 112:E862–70. 10.1073/pnas.1417683112 25675496 PMC4345584

[45] Bashford-Rogers RJ Palser AL Idris SF Carter L Epstein M Callard RE Capturing needles in haystacks: a comparison of B-cell receptor sequencing methods. BMC Immunol (2014) 15:29. 10.1186/s12865-014-0029-0 25189176 PMC4243823

[46] Lindner C Thomsen I Wahl B Ugur M Sethi MK Friedrichsen M Diversification of memory B cells drives the continuous adaptation of secretory antibodies to gut microbiota. Nat Immunol (2015) 16(8):880–8. 10.1038/ni.3213 26147688

[47] Deakin CT Deakin JJ Ginn SL Young P Humphreys D Suter CM Impact of next-generation sequencing error on analysis of barcoded plasmid libraries of known complexity and sequence. Nucleic Acids Res (2014) 42:e129. 10.1093/nar/gku607 25013183 PMC4176369

[48] Arnaout R Lee W Cahill P Honan T Sparrow T Weiand M High-resolution description of antibody heavy-chain repertoires in humans. PLoS One (2011) 6:e22365. 10.1371/journal.pone.0022365 21829618 PMC3150326

[49] Gitlin AD Shulman Z Nussenzweig MC. Clonal selection in the germinal centre by regulated proliferation and hypermutation. Nature (2014) 509:637–40. 10.1038/nature13300 24805232 PMC4271732

[50] Laydon DJ Bangham CRM Asquith B Crm B. Estimating T-cell repertoire diversity: limitations of classical estimators and a new approach. Philos Trans R Soc B (2015) 370:1–11. 10.1098/rstb.2014.0291 26150657 PMC4528489

[51] Racanelli V Brunetti C De Re V Caggiari L De Zorzi M Leone P Antibody V(h) repertoire differences between resolving and chronically evolving hepatitis C virus infections. PLoS One (2011) 6:e25606. 10.1371/journal.pone.0025606 21980500 PMC3182224

[52] Adderson E Shackelford P Quinn A Wilson P Cunningham M Insel R Restricted immunoglobulin VH usage and VDJ combinations in the human response to *Haemophilus influenzae* type b capsular polysaccharide. J Clin Invest (1993) 91:2734–43. 10.1172/JCI116514 8514881 PMC443339

[53] Liu L Lucas AH. IGH V3-23*01 and its allele V3-23*03 differ in their capacity to form the canonical human antibody combining site specific for the capsular polysaccharide of *Haemophilus influenzae* type b. Immunogenetics (2003) 55:336–8. 10.1007/s00251-003-0583-8 12845501

[54] Michaelsen TE Garred P Aase A. Human IgG subclass pattern of inducing complement-mediated cytolysis depends on antigen concentration and to a lesser extent on epitope patchiness, antibody affinity and complement concentration. Eur J Immunol (1991) 21:11–6. 10.1002/eji.1830210103 1703960

[55] Boyd SD Liu Y Wang C Martin V Dunn-Walters DK. Human lymphocyte repertoires in ageing. Curr Opin Immunol (2013) 25:511–5. 10.1016/j.coi.2013.07.007 23992996 PMC4811628

[56] Barrett DJ Ayoub EM. IgG2 subclass restriction of antibody to pneumococcal polysaccharides. Clin Exp Immunol (1986) 63:127–34. 3955880 PMC1577346

[57] Lottenbach KR Mink CM Barenkamp SJ Anderson EL Homan SM Powers DC. Age-associated differences in immunoglobulin G1 (IgG1) and IgG2 subclass antibodies to pneumococcal polysaccharides following vaccination. Infect Immun (1999) 67:4935–8. 10456954 10.1128/iai.67.9.4935-4938.1999 PMC96832

[58] Grönwall C Vas J Silverman GJ Protective roles of natural IgM antibodies. Front Immunol (2012) 3:36 10.3389/fimmu.2012.00066 22566947 PMC3341951

[59] Galili U Anaraki F Thall A Hill-Black C Radic M. One percent of human circulating B lymphocytes are capable of producing the natural anti-Gal antibody. Blood (1993) 82:2485–93. 7691263

