# Supplementary material for: In-Depth Assessment of Within-Individual and Inter-Individual Variation in the B Cell Receptor Repertoire
Source: Front Immunol. 2015 Oct 12;6:531. doi: 10.3389/fimmu.2015.00531 (PMC4601265; doi:10.3389/fimmu.2015.00531)
Supplement: Supplementary file 1 [file Data_Sheet_1.PDF]

Supplementary Material

In-depth assessment of within-individual and inter-individual variation in the B cell receptore repertoire

Jacob D. Galson*, Johannes Trück, Anna Fowler, Márton Münz, Vincenzo Cerundolo, Andrew J. Pollard, Gerton Lunter & Dominic F. Kelly

*** Correspondence:** Jacob D. Galson: jacob.galson@paediatrics.ox.ac.uk

1. Supplementary Figures 1-6

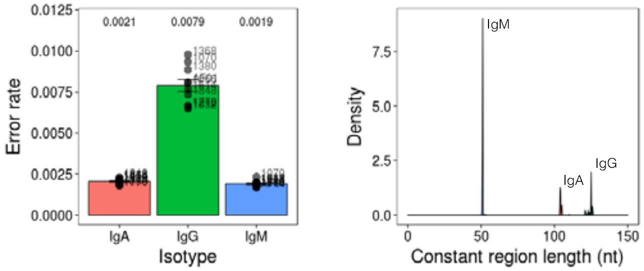

Figure S1. Error estimation. (**A**) Mean error rates of sequences obtained from each sample. Error bars shown ± SEM. For each sequence, error is calculated as number of mismatches in the constant region from germline divided by the length of the captured constant region sequence. (**B**) The length distribution of constant region sequences available for determining error of samples amplified using primers for the different isotypes.

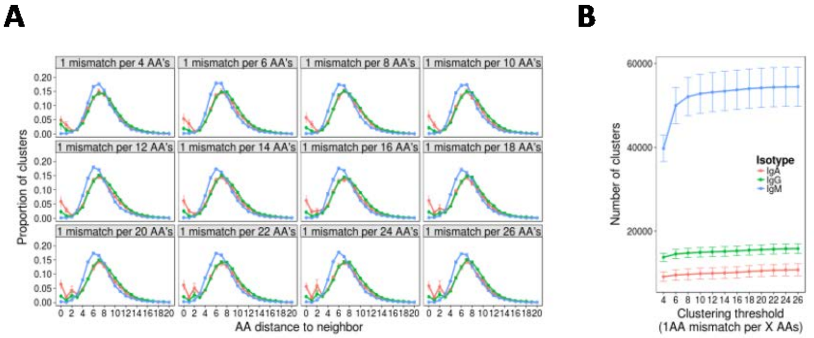

Figure S2. Trialing different clustering thresholds. (**A**) Nearest-neighbor distribution of cluster center CDR3 AA sequences after clustering with different thresholds of mismatch allowed. The nearest neighbor of each sequence in each dataset is determined by comparing it to every other sequence of the same length in the dataset to find the closest match – that is its nearest neighbor. The distance is then the number of AA difference between the sequence and its nearest neighbor. (**B**) The number of clusters formed following clustering with different thresholds of mismatch allowed. For **A** and **B**, results were obtained from samples from all 10 participants, and mean ± SEM values plotted.

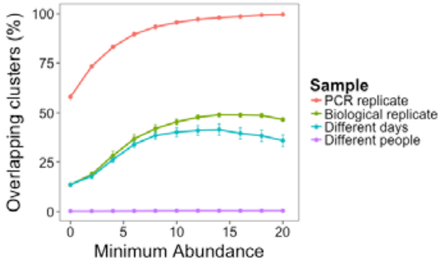

Figure S3. Overlap in clusters from different samples. The percent of common clusters present in pairs of different sample types was calculated, where percent = (A∩B/min(A,B)*100. This was determined while subsetting one of the pairs to contain only clusters above a certain abundance. Values represent mean ± SEM for comparisons between five sets of PCR replicates, five sets of biological replicates, five different days, and ten different participants.

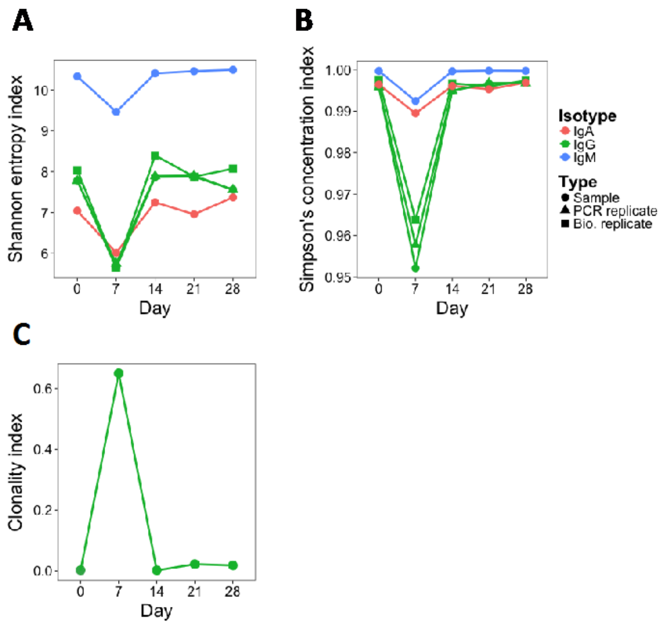

Figure S4. Comparison of single diversity/clonality indices. (**A**) Shannon's entropy and (**B**) Simpson's concentration indices calculated from all samples from participant AF01. (**C**) Clonality index calculated from IgG sample from participant AF01 – calculating this index requires biological replicates, so cannot be done for the other isotypes.

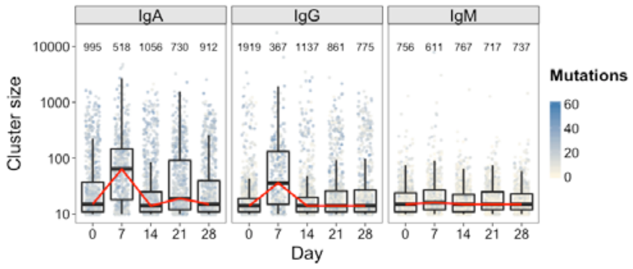

Figure S5. Size distribution of abundant clusters in participant AF01. Every abundant cluster present at each day is plotted as a point (total number is above each day), and jittered to prevent over-plotting. Boxplots show locations of 25, 50 and 75th percentiles of cluster size, and whiskers extend to maximum values that lie within 1.5x the interquartile range above and below the quartiles. Red line connects median value at each timepoint.

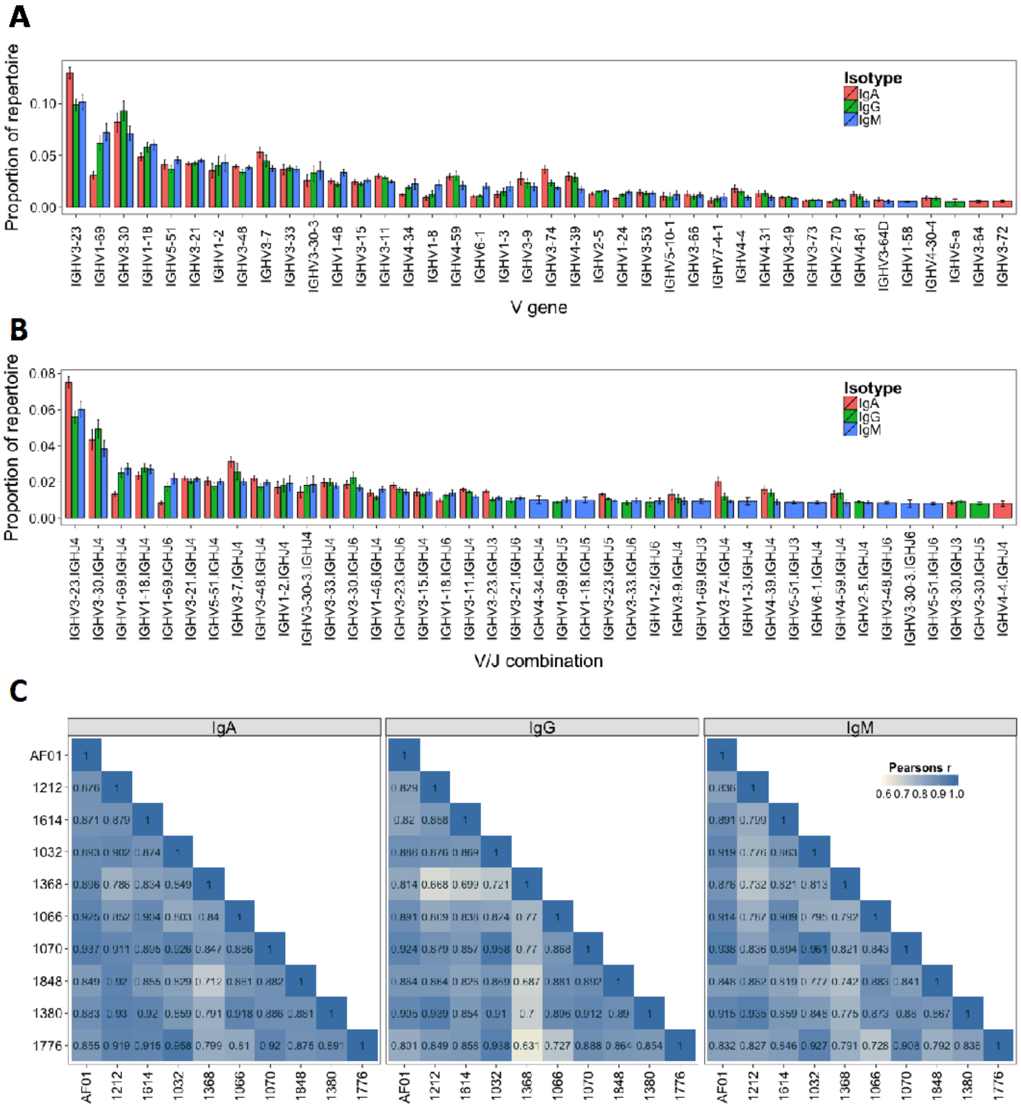

Figure S6. Uneven V and VJ use. Usage frequency of different V genes (**A**) or V/J gene combination (**B**) in the repertoire. Of the 71 different V genes, and 379 different V/J combinations, only the 41 most frequent are shown. Ordering is by frequency in the IgM dataset. Bars show mean values from 10 participants, and error bars indicate ± SEM. (**C**) Correlation in relative usage proportion of each VJ gene combination from samples from different participants. Stronger correlations are in darker blue.

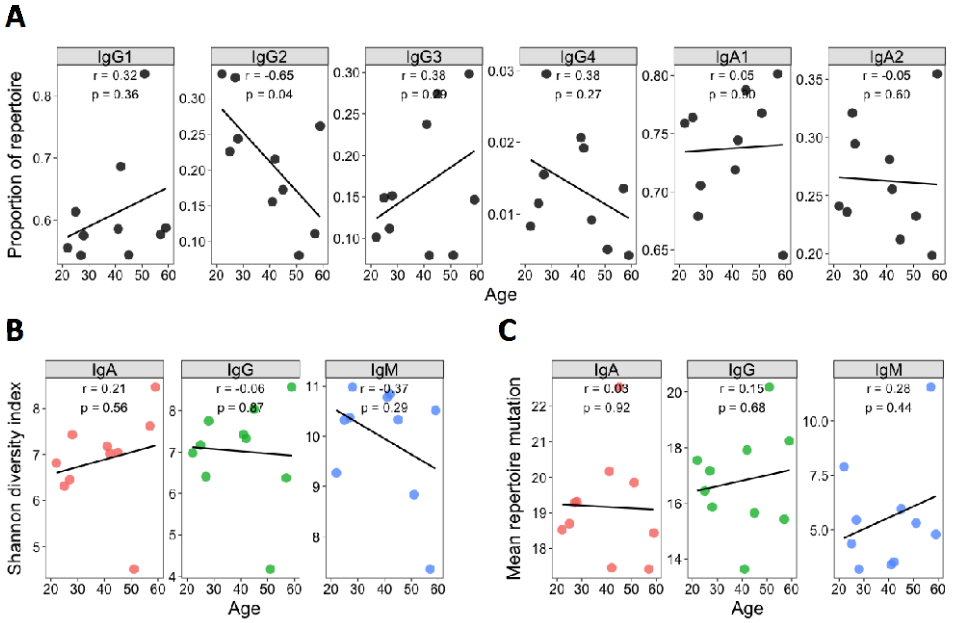

Figure S7. Correlating repertoire properties with age. (**A**) Proportion of the repertoire comprised by the different IgG and IgA subclasses, (**B**) Shannon diversity, and (**C**) mean repertoire mutation in the different aged participants. Note that the decrease in IgG2 with age is the only significant correlation ($p < 0.05$).

2. Supplementary Tables 1-3

Table S1. Summary of samples sequenced, and the number of raw sequences, filtered sequences, and clusters obtained for each sample. IgG 2 samples represent PCR replicates, and IgG 3 samples represent biological replicates.

Participant	Day	Isotype	Raw sequences	Filtered sequences	Clusters
AF01	0	IgA	369765	288434	9356
AF01	0	IgG 1	366431	233970	12152
AF01	0	IgG 2	339893	260545	12447
AF01	0	IgG 3	1516275	293725	17390
AF01	0	IgM	381663	258266	55821
AF01	7	IgA	416194	230182	7274
AF01	7	IgG 1	269345	169104	13186
AF01	7	IgG 2	394522	227356	13350
AF01	7	IgG 3	324996	215400	11812
AF01	7	IgM	300980	287619	51019
AF01	14	IgA	293845	205845	11814
AF01	14	IgG 1	406863	239804	16907
AF01	14	IgG 2	380327	223979	17679
AF01	14	IgG 3	350925	216530	23262
AF01	14	IgM	271986	239790	58103
AF01	21	IgA	310648	290743	11259
AF01	21	IgG 1	480514	294468	17930
AF01	21	IgG 2	333238	189606	18200

AF01	21	IgG 3	296139	188429	21374
AF01	21	IgM	381581	376755	59674
AF01	28	IgA	258421	312684	12506
AF01	28	IgG 1	439999	283522	15463
AF01	28	IgG 2	344963	257667	15784
AF01	28	IgG 3	464562	226976	22029
AF01	28	IgM	430162	313704	60765
1032	0	IgA	281579	217679	4946
1032	0	IgG	243879	162316	5221
1032	0	IgM	331231	280821	47051
1066	0	IgA	384924	275867	11360
1066	0	IgG	336302	225133	16560
1066	0	IgM	410635	318065	75112
1070	0	IgA	367806	256992	11684
1070	0	IgG	353992	220301	13513
1070	0	IgM	533009	414699	24623
1212	0	IgA	292080	217819	6500
1212	0	IgG	280407	216153	7278
1212	0	IgM	263810	212145	35916
1368	0	IgA	324406	254355	1658
1368	0	IgG	229626	176822	1599
1368	0	IgM	327464	275008	18998
1380	0	IgA	282185	298121	9939
1380	0	IgG	376162	185112	14116
1380	0	IgM	385190	311340	71570
1614	0	IgA	265633	204467	5825
1614	0	IgG	216878	149410	10584
1614	0	IgM	295468	245040	45118
1776	0	IgA	252253	277397	12542
1776	0	IgG	309604	249765	19061
1776	0	IgM	359614	312789	75422
1848	0	IgA	387084	277757	22942
1848	0	IgG	427766	292150	24998
1848	0	IgM	473752	370878	64903

Table S2. Immunoglobulin heavy chain V gene genotypes (determined using TIgGER) for the 10 participants in the study. Novel alleles are shown in red, and named according to TIgGER conventions. NA indicates that there were too few sequences mapped to a gene for accurate assignment of genotype.

V gene	Genotype of each participant									
	1032	1066	1070	1212	1368	1380	1614	1776	1848	AF01
IGHV1-2	4	02_T163C,04	02,02_T163C	2	02,04	4	2	2	4	2
IGHV1-3	1	1	1	NA	1	1	1	1	1	NA
IGHV1-8	1	1	1	1	1	NA	1	NA	1	1
IGHV1-18	04,01	04,01	1	1	04,01	1	01,04	04,01	04,01	1
IGHV1-24	1	1	1	1	1	1	1	1	1	1
IGHV1-46	1	01,03	1	1	1	01,01_C213G	3	03,01	1	01,03
IGHV1-58	01,02	1	01,02	01,02	2	01,02	01,02	2	01,02	01,02
IGHV1-69	04,13,06	13,06,10	02,13,10_C184T	13	02,13_C191T	02,04	13,06	02,13	13,04	13,02
IGHV2-5	NA	2	NA	1	NA	2	NA	2	2	NA
IGHV2-26	NA	NA	NA	NA	NA	NA	NA	NA	NA	NA
IGHV2-70	01,11	01,04	01,04	11,01	11	11	01,04	11,01	01,11	NA
IGHV3-7	01,03	01,03	1	1	01,03	3	01,03	3	03,01	1
IGHV3-9	1	1	1	1	1	NA	1	NA	1	1
IGHV3-11	01,06	01,06	03,01	1	01,06	03,06	01,06	6	06,01	1
IGHV3-13	01,05	NA	04,01	1	1	04,05	1	NA	NA	1
IGHV3-15	1	1	1	1	1	07,01	1	01,07	1	NA

IGHV3-20	1	NA	NA	1	1	01_C307T	1	NA	NA	1
IGHV3-21	1	1	1	1	1	1	1	1	1	1
IGHV3-23	1	1	1	1	1	1	1	1	1	1
IGHV3-30	18	18	03,18	18	03,18	4	18	03,18,02	18	18
IGHV3-30-3	1	1	NA	1	NA	NA	1	1	1	1
IGHV3-33	1	1	1	1	01,06	01_G75C	1	1	1	1
IGHV3-43	1	NA	1	1	1	1	1	01,01	1	1
IGHV3-48	01,02	03,01	04,02	02,01	02,01	01,04	03,01	02,01	2	02,01
IGHV3-49	NA	03,04	NA	3	NA	3	NA	04,03	3	NA
IGHV3-53	1	1	01,04	1	1	01,04	1	04,01	1	1
IGHV3-64	06_T70G,01	NA	1	NA	1	1	1	1	06_T70G,01	NA
IGHV3-66	1	NA	2	1	2	02,01	NA	1	1	2
IGHV3-72	NA	NA	NA	NA	NA	NA	NA	NA	NA	NA
IGHV3-73	NA	1	NA	NA	NA	NA	NA	NA	1	1
IGHV3-74	2	2	2	2	2	2	2	2	2	2
IGHV4-4	07,02	5	07,05	7	07,02,05	7	07,05	7	05,02	7
IGHV4-30-2	NA	NA	NA	NA	NA	NA	NA	NA	NA	NA
IGHV4-30-4	1	NA	NA	1	NA	NA	1	NA	1	1
IGHV4-31	4	4	4	4	4	NA	4	4	4	4
IGHV4-34	1	1	1	1	1	1	1	1	1	1
IGHV4-39	1	01_G298A	07_C288A,01	1	1	07_C288A,01	1	01,07	1	1
IGHV4-59	01,08,07	01,07	01,08,07	01,07	01,08	01,08,07	01,08	01,08,07	01,07,08	01,07
IGHV4-61	1	1	NA	1	2	1	1	02_A234G	NA	02,01
IGHV5-51	3	03,01	1	01,03	3	1	3	03,01	1	03,01
IGHV5-10-1	1	1	1	NA	1	1	1	1	1	NA
IGHV6-1	1	1	1	1	1	1	1	1	1	1
IGHV7-4-1	NA	2	2	NA	NA	2	2	2	1	NA

Table S3. Antigen-specific sequences collated from the literature that map to clusters in our dataset.

Antigen	Subtype	CDRH3 AA sequence	Reference
TT	NA	YTTDLPTVGAGEFDYW	*(27)*
TT	NA	CTTDLPTVGAGEFDYW	*(27)*
TT	NA	CTTDLPTVGAGECDYW	*(27)*
TT	NA	CATGVTLDYW	*(27)*
TT	NA	CATGITLDYW	*(27)*
TT	NA	CATGFTLDYW	*(27)*
TT	NA	CARVRSGTYDYW	*(27)*
TT	NA	CARVLSGTYDYW	*(27)*
TT	NA	CARVLSGTNDYW	*(27)*
TT	NA	CARVLSGTCDYW	*(27)*
TT	NA	CARVLSGSYDYW	*(27)*
TT	NA	CARVLSGPYDYW	*(27)*
TT	NA	CARVLSGNYDYW	*(27)*
TT	NA	CARVLSGIYDYW	*(27)*
TT	NA	CARVLSGAYDYW	*(27)*
TT	NA	CARGVVPAGTPFDFW	*(27)*
TT	NA	CARGLSGTYDYW	*(27)*
TT	NA	CASTRSSTWYLDYW	*(28)*
TT	NA	CARQTDNWFDPW	*(28)*
TT	NA	CALTYDNWFDPW	*(28)*
TT	NA	CASGSTLDYW	*(30)*
TT	NA	CARDYYGSGSHYYFDYW	*(30)*
TT	NA	CARRYDFWSGFLDYW	*(30)*
TT	NA	CARRHYCSSTSCYDAFDIW	*(30)*
TT	NA	CVSGGSLDYW	*(28)*
TT	NA	CATGRTLDYW	*(28)*
TT	NA	CATGNTLDYW	*(28)*
TT	NA	CASGYCSSTSCYDYW	*(28)*
TT	NA	CASGVTHDYW	*(28)*

TT	NA	CASGSTLDYW	*(28)*
TT	NA	CASGRSLDYW	*(28)*
TT	NA	CARWRWHQSEFDYW	*(28)*
TT	NA	CARTVRGVVPFDYW	*(28)*
TT	NA	CARTVGVVLPFDYW	*(28)*
TT	NA	CARSVVPATRSFDYW	*(28)*
TT	NA	CARRYDFWSGFLDYW	*(28)*
TT	NA	CARRHYCSSTSCYDAFDIW	*(28)*
TT	NA	CARQTDNWFDPW	*(28)*
TT	NA	CARLGLIW	*(28)*
TT	NA	CARGGSSAFDVW	*(28)*
TT	NA	CAREYGDYKFDYW	*(28)*
TT	NA	CAREEFTSSSRWFDPW	*(28)*
TT	NA	CARDYYGSGSHYYFDYW	*(28)*
TT	NA	CARDYSSPYYFDYW	*(28)*
TT	NA	CARDYFGSGSVYYFDYW	*(28)*
TT	NA	CARDYFGSGSIYYFDYW	*(28)*
TT	NA	CARDHSSPYYFDYW	*(28)*
TT	NA	CARDFYSGSYRSFDYW	*(28)*
TT	NA	CAKDRQLKDAFDIW	*(28)*
TT	NA	CAKDLYGDYDLDYW	*(28)*
Influenza	H2N2/H3N2	CARLYGSGLDYW	*(31)*
Influenza	H2N2/H3N2	CARGISGSYGWFDPW	*(31)*
Influenza	H1N1	CARHGYGDYVGYFDYW	*(32)*
Influenza	H1N1	CARGSTYYSSYFDQW	*(32)*
Influenza	H1N1	CARSGSYYPDYFQYW	*(32)*
Influenza	H1N1	CARAPLIYNWYFDLW	*(32)*
Influenza	cross-reactive	CASRYSSGWYYFDYW	*(33)*
Influenza	cross-reactive	CASGRDFYYYGMDVW	*(33)*
Influenza	cross-reactive	CARILSADYYYGMDVW	*(33)*
Influenza	cross-reactive	CARHYGGYDWFDPW	*(33)*
Influenza	cross-reactive	CAREEGTYYDFWSANNWFDPW	*(33)*
Influenza	cross-reactive	CAKRYSSGWYSFDYW	*(33)*
Influenza	cross-reactive	CAKGSGYHVRDYFDYW	*(33)*
Influenza	cross-reactive	CARGNYYYESSLDYW	Patent: US 8,192,927
Influenza	cross-reactive	CARGPHYYSSYMDVW	Patent: US 8,192,927
Influenza	cross-reactive	CARGPTYYYSYMDVW	Patent: US 8,192,927
Influenza	cross-reactive	CARGLYYYESSLDYW	Patent: US2011/0038935
Influenza	cross-reactive	CASSSGSYYGDYFDYW	Patent: US2012/096994
Influenza	H1N1	CARHDSSGYHPLDYW	*(34)*
